# *Thymus mastichina*: Composition and Biological Properties with a Focus on Antimicrobial Activity

**DOI:** 10.3390/ph13120479

**Published:** 2020-12-19

**Authors:** Márcio Rodrigues, Ana Clara Lopes, Filipa Vaz, Melanie Filipe, Gilberto Alves, Maximiano P. Ribeiro, Paula Coutinho, André R. T. S. Araujo

**Affiliations:** 1 School of Health Sciences, Polytechnic Institute of Guarda, Rua da Cadeia, 6300-035 Guarda, Portugal; claralopes28@gmail.com (A.C.L.); filipa.a.c.vaz@hotmail.com (F.V.); melaniemfilipe@gmail.com (M.F.); mribeiro@ipg.pt (M.P.R.); 2 Research Unit for Inland Development (UDI), Polytechnic Institute of Guarda, Av. Dr. Francisco Sá Carneiro, 50, 6300-559 Guarda, Portugal; 3 CICS-UBI—Health Sciences Research Centre, University of Beira Interior, Av. Infante D. Henrique, 6200-506 Covilhã, Portugal; gilberto@fcsaude.ubi.pt; 4 LAQV/REQUIMTE, Department of Chemical Sciences, Faculty of Pharmacy, University of Porto, Rua Jorge Viterbo Ferreira, 228, 4050-313 Porto, Portugal

**Keywords:** antimicrobial, biological activities, essential oil, extract, *Thymus mastichina*

## Abstract

*Thymus mastichina* has the appearance of a semishrub and can be found in jungles and rocky lands of the Iberian Peninsula. This work aimed to review and gather available scientific information on the composition and biological properties of *T. mastichina*. The main constituents of *T. mastichina* essential oil are 1,8-cineole (or eucalyptol) and linalool, while the extracts are characterized by the presence of flavonoids, phenolic acids, and terpenes. The essential oil and extracts of *T. mastichina* have demonstrated a wide diversity of biological activities. They showed antibacterial activity against several bacteria such as *Escherichia coli*, *Proteus mirabilis*, *Salmonella* subsp., methicillin-resistant and methicillin-sensitive *Staphylococcus aureus*, *Listeria monocytogenes EGD*, *Bacillus cereus*, and *Pseudomonas*, among others, and antifungal activity against *Candida* spp. and *Fusarium* spp. Additionally, it has antioxidant activity, which has been evaluated through different methods. Furthermore, other activities have also been studied, such as anticancer, antiviral, insecticidal, repellent, anti-Alzheimer, and anti-inflammatory activity. In conclusion, considering the biological activities reported for the essential oil and extracts of *T. mastichina*, its potential as a preservative agent could be explored to be used in the food, cosmetic, or pharmaceutical industries.

## 1. Introduction

**Figure 1:**
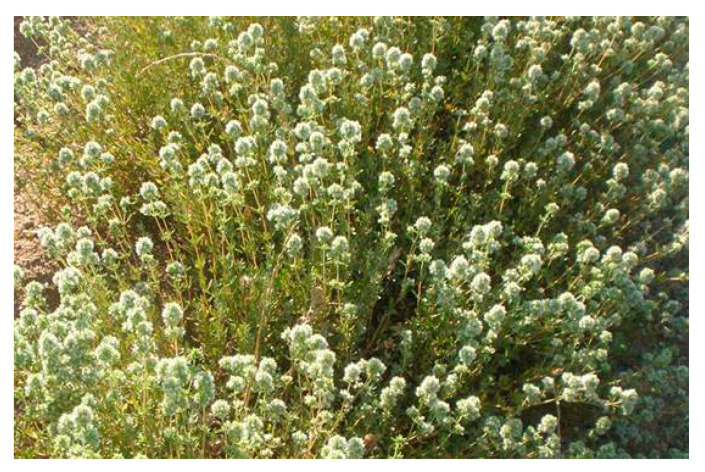
*Thymus mastichina* plant (source Planalto Dourado^TM^ Essential Oils Enterprise, from Freixedas, Guarda, Portugal).

*Thymus mastichina* L. (Figure 1) is an endemic species of the Iberian Peninsula, commonly known as “Bela-Luz”, “Sal-Puro”, “Tomilho-alvadio-do-Algarve”, “Mastic thyme”, and “Spanish marjoram” and belongs to the Lamiaceae family [1,2,3,4]. *T. mastichina* species can be classified into two subspecies: *T. mastichina* subsp. *donyanae* and *T. mastichina* subsp. *mastichina*; the first of which is present in Algarve (Portugal) and Huelva and Seville (Spain) and the latter extends throughout the Iberian Peninsula [5,6]. This aromatic plant is a semiwoody shrub that grows up to 50 cm tall and is characterized by simple and opposite leaves and bilabiate flower groups in a flower head or capitula, which blossom from April to June [2,7,8]. *T. mastichina* can be found in jungles, uncultivated, ruderal, and rupicolous lands and in dry stony open places, except in calcareous regions [1,8], being very resistant to frost, diseases, and pests. *T. mastichina* is known for its strong eucalyptus odor and it has been used for various health conditions due to its antiseptic, digestive, antirheumatic, and antitussive effects [2,6,9,10].

*T. mastichina* can be used in fresh or dry form and its leaves are traditionally used as a condiment/spices flavoring, in seasoning traditional dishes and salads, to preserve olives, to aromatize olive oil, and as a substitute for salt [10,11]. This medicinal and aromatic plant is also used as a source of essential oil in the cosmetic and perfume industries [10,12]. Infusions with dry parts of the plant have been used to relieve colds, cough, throat irritations, and abdominal pain, while infusions with fresh parts of the plant have been used for indigestion and stomach pain [13]. Thus, there are different products based on *T. mastichina* that are commercially available in Portugal and Spain (e.g., “Bela-Luz” essential oil, “Marjoram Spanish” essential oil, “Tomilho Bela Luz” herbs). In this work, we compiled the available information regarding the chemical composition of the essential oil and extracts of *T. mastichina* to review published studies in which its biological activities have been evaluated.

## 2. Search Strategy

The search was performed on the following databases: PubMed and Web of Science. Various combinations of the following terms were queried: *T. mastichina*, thyme, antibacterial, antifungal, antimicrobial, anti-inflammatory, anti-Alzheimer, antioxidant, anticancer, antiviral, insecticidal, repellent, essential oil, plant extract, biological activity, and composition. In addition, references cited in related publications were followed up. The selection of articles was performed by its relevance to the purpose of this review. It should be noted that no date or language criteria were defined as filters in the search strategy implemented.

## 3. Chemical Composition of *T. mastichina* Essential Oils and Extracts

The essential oil of *T. mastichina* is usually obtained mainly by hydrodistillation for 2–4 h, with low yields (from 0.4% to 6.90% (*v*/*w*)) (Table 1). Other extraction methods were also used, such as microdistillation, that showed higher amounts of 1,8-cineole plus limonene in comparison with hydrodistillation. However, in general, few differences were found between the different methodologies. The yield may vary depending on several factors, namely the part of the plant used, place of harvest, period of the year, storage time, extraction time, and type of fertilization, among others [14].

**Figure 2:**
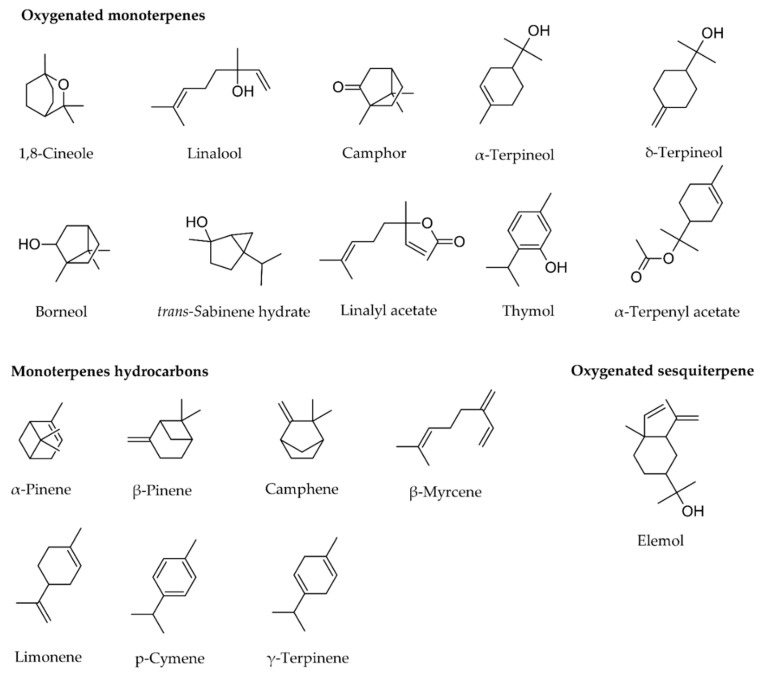
Chemical structures of main constituents of the *Thymus mastichina* essential oil categorized in oxygenated and hydrocarbons monoterpenes and hydrocarbon sesquiterpene.

As shown in Figure 2 and Table 1, the composition of the essential oil of *T. mastichina* is quite diverse, being constituted by hydrocarbon and oxygenated monoterpenes and sesquiterpenes, presenting main constituents of 1,8-cineole (also known as eucalyptol) and linalool. The major constituents of the essential oil of *T. mastichina* have been determined by gas chromatography [2,4,8,14,15,16,17,18,19,20,23,24,25,26,27,28,29,30,31,32,34,35,36,37,38,39,40,41,42,43,44,45,47,48]. Table 1 summarizes the major constituents present and it can be seen that their composition varies according to origin. In fact, there are three main subtypes of essential oils, depending on the main compounds present: 1,8-cineole, linalool, and 1,8-cineole/linalool. In Portugal, the chemotypes of linalool and 1,8-cineole/linalool are only found in Estremadura, mainly at Arrábida and Sesimbra, while the 1,8-cineole chemotype is distributed throughout the country [8,21]. As expected, there are differences in the phytochemical composition among different species of Thymus; for instance, the main compound presented in *Thymus vulgaris* is thymol, which is present in low percentages in *T. mastichina* [49]. 

As presented in Table S1, in addition to *T. mastichina* essential oil, in some cases after hydrodistillation, the decoction water (the remaining hydrodistillation aqueous phase) was also collected [12,14,32,43,50]. Conversely, in some studies, extraction from the aerial parts of the plant was performed using different solvents (hexane, dichloromethane, ethanol, diethyl ether, ethyl acetate, *n*-butanol, and water) [10,13,32,37,43,50,51]. Furthermore, in some cases, the extracts were obtained by ultrasound [9,51]. The extracts obtained from the aerial parts of the plant were characterized by the presence of different polyphenol classes, in particular, flavonoids (apigenin, kaempferol, luteolin, naringenin, quercetin, sakuranetin, sterubin), phenolic acids (caffeic acid, chlorogenic acid, 2-methoxysalicylic acid, 3-methoxysalicylic acid, rosmarinic acid, salvianolic acids I and K and derivatives), phenolic terpene (carnosol) and hexoside and glycoside derivatives; other compounds identified were steroid (β-sitosterol), triterpenoids (oleanolic acid, ursolic acid), and xanthophyll lutein [9,10,12,37,51,52]. In Figure S1 the chemical structures of several compounds present in the extract are presented. In opposition to *T. mastichina* essential oil, in which the composition is extensively characterized, the extract phenolic profile has been less investigated and diverse chromatographic peaks detected during the phytochemical characterization remain unidentified. 

## 4. Biological Properties

The diverse bioactivities of *T. mastichina* essential oil and extracts are related to the chemical composition. Essential oil and extracts from the aerial parts of *T. mastichina* have been mainly described for their antibacterial and antifungal activities, but also for antioxidant, anticancer, antiviral, insecticidal, insect repellent, and anti-enzymatic activities (anti-Alzheimer, anti-inflammatory, α-amylase, and α-glucosidase). 

### 4.1. Antibacterial and Antifungal Activities

The antibacterial and antifungal activities of the essential oils and their main compounds were evaluated by several researchers. The effect of antibacterial activity of essential oils may inhibit the growth of bacteria (bacteriostatic) or destroy bacterial cells (bactericidal). Nevertheless, it is difficult to distinguish these actions, as the antibacterial activity evaluation if frequently based on the most known and basic methods such as disk-diffusion and broth microdilution for the determination of the diameter of the zone of inhibition, minimum inhibitory concentration (MIC), and minimum lethal concentration (MLC), and mixtures of methods, as can be seen in Table 2. 

The antimicrobial activity of *T. mastichina* essential oil from the chemotype of Algarve and two chemotypes of Sesimbra (Estremadura) in Portugal, measured by disc agar diffusion method, was confirmed by Faleiro et al. [21]. The tested microorganisms (*Escherichia coli*, *Proteus mirabilis*, *Salmonella* subsp., *Staphylococcus aureus*, *Listeria monocytogenes EGD*) presented different sensitivities. In particular, *T. mastichina* essential oil (3 μL) from Algarve showed the highest activity against *S. aureus* showing a diameter of the zone of inhibition of 13.7 and 15.7 mm for the flower and leaf, respectively, while *T. mastichina* essential oil from Sesimbra (Estremadura) had the highest activity for *S. aureus* showing a diameter of the zone of inhibition of 13.3 mm for chemotype A. Additionally, the antimicrobial activity observed with *T. mastichina* essential oil was explored to determine if it was due to the main constituents present in the different oil chemotypes (linalool, 1,8-cineole and linalool/1,8-cineole (1:1) mixture); for this purpose, the antimicrobial activity of these constituents alone was tested. It was concluded a higher antimicrobial activity of linalool compared with 1,8-cineole. Moreover, possible antagonist and synergistic effects of the various constituents of the essential oil were registered, once *E. coli* was susceptible to linalool but not to the mixture of linalool plus 1,8-cineole, and for *C. albicans* the mixture of linalool plus 1,8-cineole produced a slight increase in the antimicrobial activity whereas was not susceptible to 1,8-cineole. In a previous work of the same authors, and using the same method, the results suggested that the higher antimicrobial activity of the *T. mastichina* essential oil against *Salmonella* was associated to higher amounts of camphor present in *T. mastichina* essential oil comparatively to the essential oils of other plants [16].

*T. mastichina* essential oils with origin in different bioclimatic zones from Murcia (Spain) showed activity (growth inhibition) against Gram-positive (methicillin-sensitive *S. aureus*), Gram-negative (*E. coli*), and fungi (*C. albicans*). However, some differences were identified among them, probably due to the influence of the clime in the composition of the essential oil. In particular, *T. mastichina* essential oil from the Supra-Mediterranean bioclimatic zone (Moratalla, Spain) produced higher inhibition against *C. albicans* than the *T. mastichina* essential oils from other bioclimatic zones (Caravaca de la Cruz and Lorca, Spain) due to the high concentration of linalool [4]. However, other studies reported lower antibacterial activities of *T. mastichina* essential oil than those found in this study [29,45]. Recently Arantes et al. [2] described the *T. mastichina* essential oil broad spectrum of antibacterial activity against several strains. It was observed higher susceptibility (lower MICs) observed in Gram-negative bacteria (*E. coli*, *Morganella morganii*, *P. mirabilis*, *Salmonella enteritidis*, *Salmonella typhimurium*, and *Pseudomonas aeruginosa*) than in Gram-positive ones (methicillin-sensitive *S. aureus*, *Staphylococcus epidermidis*, and *Enterococcus faecalis*) for both agar disc diffusion assay and MICs determination by broth microdilution assay. The higher antibacterial activity against Gram-negative is suggested to be correlated with the presence of monoterpene and phenolic compounds capable of disintegrating the outer membrane of Gram-negative strains. In fact, essential oils are characterized by unique antibacterial potential due to the high number of chemical compounds present in their composition, which act simultaneously, preventing resistance mechanisms in bacteria. Furthermore, synergistic interactions between compounds of essential oils can potentiate their natural antimicrobial effect. Thus, antimicrobial potential cannot be associated with only one component or mechanism of action. Nevertheless, due to the lipophilic character of essential oils, the mechanism of action could be related to the alteration of cell membrane properties. 

The antifungal activity of *T. mastichina* essential oils from Sesimbra (Estremadura, Portugal) was observed against *C. albicans* [21]. The antifungal capacity of the *T. mastichina* essential oil against *Candida* spp. have also been evaluated through the macrodilution method that enables the determination of MIC and MLC [26]. Flow cytometry was also used as a complementary method for the study of the mechanisms responsible for antifungal activity. *T. mastichina* essential oil showed higher inhibition compared to the other *Thymus* species tested, with a lower MIC concentration obtained for *T. mastichina* against *Candida* spp. varied from 1.25 to 10.00 μL/mL, depending on the species of *Candida*, while the MLC remained at 5 μL/mL for almost all species. This study described the potent antifungal activity of *T. mastichina* essential oil against *Candida* spp., warranting future therapeutic trials on mucocutaneous candidosis. Compared with other studies, similar MIC values for *Candida* were found using *T. mastichina* essential oil from Portugal [4]. A remarkable increase in antifungal activity of the mixture of extracts of *Rosmarinus officinalis*, *Salvia lavandulifolia*, *T. mastichina*, and chitosan against different yeasts (*C. albicans*, *Pichia anomala*, *Pichia membranaefaciens*, and *Saccharomyces cerevisiae*) and filamentous fungi (*Aspergillus niger* and *Penicillium digitatum* strains belonging to the collection of fungi isolated from citrus) was observed [53]. However, the results obtained did not enable the determination of MICs or minimum fungicidal concentrations (MFCs) because the values were higher than the maximum concentrations tested. Additionally, the fungicide activity of *T. mastichina* extracts (at 20–25 mg/mL) from plants micropropagated in vitro against *Aspergillus fumigatus* was demonstrated for the first time [56]. In another study, the antifungal capacity of *T. mastichina* essential oil was determined against *Fusarium* spp. using the agar dilution method. *T. mastichina* essential oil showed antifungal activity against pathogenic fungi strains of the genus *Fusarium* with MICs and MFCs ranging from 1500 to 2100 μg/mL and from 2.0 to 2.4 mg/mL, respectively. In this study, the antifungal activity of the two main constituents, 1,8-cineole and linalool, was also evaluated and considering the obtained MICs and MFCs, the antifungal activity of the essential oil seemed to be due to the presence of major constituents [41].

On the other hand, the potential use of *T. mastichina* essential oil as an active and functional ingredient in food products, and the antimicrobial activity against zoonotic and food spoilers and foodborne microorganisms was evaluated in several studies. 

The effect of *T. mastichina* essential oil was evaluated on several bacteria of the Enterobacteriaceae family (*E. coli, Salmonella* spp.) with an origin in poultry and pigs species, was registered with MIC values with 4% (*v*/*v*) [29], as well as in other studies against methicillin-sensitive *S. aureus* and *S. epidermidis* isolates from ovine mastitic milk [48].

Gram-negative bacteria (*E. coli*, *Salmonella enterica*, and *Enterobacter aerogenes*) and Gram-positive bacteria (*Bacillus cereus* and methicillin-resistant *S. aureus*) were used for determination of MIC, through dilution and microdilution techniques, of a mixture of extracts of *Rosmarinus officinalis*, *Salvia lavandulifolia*, *T. mastichina*, and chitosan [53]. All tested extracts demonstrated noticeable antimicrobial activities against spoilage and foodborne pathogens such as *B. cereus*, methicillin-resistant *S. aureus*, *E. coli*, *E. aerogenes*, *S. enterica*, and yeast-like fungi, without interference in sensory properties. Vieira et al. [45] also observed *T. mastichina* essential oil activity, with MIC values of 15 mg/mL (*Bacillus subtilis* and *E. coli*) and 20 mg/mL (methicillin-sensitive *S. aureus* and *P. aeruginosa*); and the same pattern for MLC, with a value of 40 mg/mL for *S. aureus*, 30 mg/mL for *B. subtilis* and *E. coli*, and 70 mg/mL for *P. aeruginosa*, and suggesting this aromatic plant to be used in control pathogenic microorganisms in deteriorating foods. In another study, the antibacterial activity of *T. mastichina* essential oil was assayed in vitro by a microdilution method against both Gram-positive (*Listeria innocua*, methicillin-resistant *S. aureus*, and *B. cereus*) and Gram-negative bacteria (*S. enterica* and *E. coli*). In this study, the inhibition percentage increased with the *T. mastichina* essential oil concentration and higher inhibition was observed for *L. innocua* [44]. Furthermore, the antimicrobial activity of whey protein isolate-based edible films incorporated with *T. mastichina* essential oil were tested against Gram-positive bacteria (*L. innocua* and methicillin-resistant *S. aureus*) and Gram-negative bacteria (*S. enteritidis* and *Pseudomonas fragi*) to be useful as a coating in the food industry. In this context, it should be highlighted that the antimicrobial activity was only observed against *L. innocua* and using the whey protein films containing 7% and 8% of *T. mastichina* essential oil [34]. This work suggests the possibility of using films incorporating essential oils on food systems. 

The active antimicrobial activity of *T. mastichina* essential oil (30 μL) applied through the disk-diffusion method was confirmed by Ballester-Costa et al. [35] against *L. innocua* and *Alcaligenes faecalis*, *Serratia marcescens*, *Enterobacter amnigenus*, and *Enterobacter gergoviae*, but not active against *P. fragi, Pseudomonas fluorescens*, *Aeromonas hydrophila*, *Shewanella putrefaciens*, *Achromobacter denitrificans*, and *E. gergoviae*. Additionally, MIC of *T. matichina* essential oil determined by microdilution assay was between 3.75 and 7.5 μL/mL for all strains. The differences obtained in both methods are related to the lower dispersion of the essential oils on a solid medium and consequently reduced ability to access to the microorganism in the disk diffusion method, confirming the unreliability of this method for essential oil evaluation. Posteriorly, the same authors tested the effect of chitosan edible film disks incorporated with the essential oil of *T. mastichina* at concentrations of 1% and 2% against *L. innocua, S. marcescens*, *E. amnigenus*, and *A. faecalis*. At both concentrations, antibacterial activity was observed for *S. marcescens*, *L. innocua*, and *A. faecalis*, with activity against *S. marcescens* being higher. However, antibacterial activity against *E. amnigenus* was not registered [54]. Besides, in the following year, they evaluated the activity of *T. mastichina* essential oil (30 μL), applied through the disk-diffusion method, against the bacteria *A. denitrificans*, *A. faecalis*, *A. hydrophila*, *E. amnigenus*, *E. gergoviae*, *L. innocua*, *P. fluorescens*, *P. fragi, S. marcescens*, and *S. putrefaciens* using, as culture medium, extracts from meat homogenates (minced beef, cooked Ham, or dry-cured sausage). *T. mastichina* essential oils were extremely active against *L. innocua* in minced beef and active for *A. hydrophila* in dry-cured sausage, while for the remaining bacteria only moderate activity or absence of activity was found [55]. In this way, it was suggested the use of *T. mastichina* essential oil as a “green” preservative agent in the food industry, per se or incorporated in edible films. In addition, it should be highlighted that its efficacy as an antibacterial agent has been demonstrated in model systems that closely simulate food composition. 

Due to the serious damage caused by fungal pathogens of agricultural interest, the possible future application of the essential oils as alternative antifungal agents was evaluated by [47]. In this study, *T. mastichina* essential oil showed either partial or complete antifungal activity against plant and mushroom pathogenic fungi (*Sclerotinia sclerotiorum*, *Fusarium oxysporum*, *Phytophthora parasitica*, *Alternaria brassicae*, and *Cladobotryum mycophilum*) by the disk-diffusion assay; the inhibitory effect of essential oils was dose-dependent on the eight tested fungi enabling the determination of ED_50_ values for most of them.

Rapid antibacterial screening of essential oils using the agar diffusion technique is usually conducted. However, the lack of standardized methods makes direct comparison of results between studies difficult [57]. The problems related to oils dispersion and lipophilic constituents in aqueous media, and varying methods for determining numbers of viable bacteria remaining after the addition of the oil were the main causes of unreliability and inconsistent results obtained from disc diffusion, well diffusion, and agar dilution methods. Nevertheless, the broth dilution method, using emulsifier, seems to be the most accurate method for testing the antimicrobial activity of the hydrophobic and viscous essential oils.

### 4.2. Antioxidant Activity

The use of antioxidants is useful in the food industry to avoid rancidity and/or deterioration of foods [58] and also to prevent reactive oxygen species (ROS) formation, such as superoxide anion, hydrogen peroxide, and hydroxyl radicals. The ROS are capable of inducing lipid peroxidation, which may result in damage of membranes, lipids, lipoproteins, and induce DNA mutations that are linked to several diseases such as rheumatoid arthritis, atherosclerosis, ischemia, carcinogenesis, and aging [31]. The antioxidant properties of different *T. mastichina* plant extracts, essential oils, and pure compounds have been evaluated using a quite diverse number of in vitro assays, namely, those that evaluate lipid peroxidation, free radical scavenging ability, and chelating metal ions. In a study conducted by Miguel et al. [22], *T. mastichina* essential oil, as well as its main constituents (1,8-cineole and linalool), evaluated through the peroxide values expressed as percentage of inhibition, showed antioxidant activity higher than that shown by the synthetic antioxidant butylated hydroxytoluene (BHT). Nevertheless, the results cannot be explained only by some of its constituents because there may be synergistic or antagonistic effects among them. Due to the demonstrated antioxidant activity, essential oils of this species seem to be a good alternative to some synthetic antioxidants. Miguel et al. [25] conducted another study, in which *T. mastichina* essential oil was tested by a modified thiobarbituric acid-reactive substances (TBARS) assay in which the antioxidant capacity was evaluated measuring the ability to inhibit lipid peroxidation. In this modified TBARS assay egg yolk was used (as a lipid-rich medium) in the presence or absence of the radical inducer of lipid peroxidation, 2,2′-azobis (2 amidinopropane) dihydrochloride (ABAP). At concentrations of 62.5–500 mg/L of essential oil, the antioxidant capacity in the absence of the peroxyl radical inducer ABAP, presented values between 9.6% and 38.9% and in the presence of the peroxyl radical inducer, ABAP lower values were achieved (−19.5–16.0%). In 2007, the same research group compared the antioxidant activity of *T. mastichina* essential oils, over a concentration range (160–1000 mg/L), isolated from different populations. The highest differences in the antioxidant activities of these essential oils were observed at the lowest concentration tested (160 mg/L), in which the *T. mastichina* essential oil from Mértola showed the lowest activity (20%), whereas *T. mastichina* essential oil from Sesimbra exhibited the highest activity (42%). Similarly, for the highest concentration tested (1000 mg/L), *T. mastichina* essential oil from Mértola showed an antioxidant index of 59% and *T. mastichina* essential oil from Sesimbra presented an inhibition percentage of 79%. At a concentration of 1000 mg/L, the *T. mastichina* essential oils from Vila Real de Santo António and Sesimbra showed a higher ability to inhibit lipid oxidation than α-tocopherol and were within the same range of activity of BHA. In comparison with assays without ABAP, the presence of the radical inducer reduced the ability of *T. mastichina* essential oils to prevent oxidation, particularly at concentrations of 160, 800, and 1000 mg/L [30]. 

In a study conducted by Galego et al. [31], the antioxidant activity of *T. mastichina* essential oil was determined using different methods, such as TBARS, measuring the scavenging effect of the substances on the 2,2-diphenyl-1-picrylhydrazyl (DPPH) radicals, determining the ferric reducing antioxidant power (FRAP) based on the principle that substances, which have reduction potential, react with potassium ferricyanide (Fe^3+^) to form potassium ferrocyanide (Fe^2+^), which then reacts with ferric chloride to form ferric–ferrous complex, and also monitoring the chelating effect on ferrous ions (Fe^2+^). The results showed that even at higher concentrations of *T. mastichina* essential oil (1000 mg/L), the antioxidant index was around 50% as observed with the TBARS method, while for the synthetic antioxidants, BHA and BHT, the antioxidant index was approximately 100%. In other methods, the difference between the activity of the essential oil and synthetic antioxidants BHA and BHT was more accentuated showing lesser antioxidant activity. 

According to Bentes et al. [32], the antioxidant activities of *T. mastichina* essential oil were screened by five different methods: DPPH free radical scavenging, modified TBARS (using egg yolk as a lipid-rich medium) for measuring the inhibition of lipid peroxidation, FRAP assay based on the reduction of ferric iron (Fe^3+^) to ferrous iron (Fe^2+^) by antioxidants present in the samples, chelating activity on ferrous ions (Fe^2+^), and superoxide anion radical scavenging. With the DPPH free radical scavenging assay, *T. mastichina* essential oil was almost totally ineffective as an antioxidant, in contrast to the remaining water-soluble hydrodistillation-aqueous phase extract that showed similar activity to that of α-tocopherol, particularly at higher concentrations (75 and 100 mg/L). This result suggests that a considerable portion of the antioxidant compounds were retained in the remaining hydrodistillation-aqueous water. In this study, the FRAP assay of *T. mastichina* essential oil was also compared. However, the correlation between DPPH and the FRAP assay activities was not as clear in the evaluation of different extracts. Whereas the hydrodistillation-aqueous phase extracts were the best antioxidants when evaluated by DPPH, the *T. mastichina* methanolic extract had a greater capacity for reducing Fe^3+^ than the hydrodistillation-aqueous phase extract. In addition, in the superoxide anion assay, only the hydrodistillation-aqueous phase and methanolic extracts were able to scavenge superoxide radicals, but the first ones were the most effective. The superoxide radical scavenging capacity of these extracts was even higher than that of the positive control (ascorbic acid, particularly at 500 mg/L). *T. mastichina* methanol extracts were powerful superoxide radical scavengers with 40–60% activity.

To determine the antioxidant activity in plant extracts, four different methods were used: the DPPH radical scavenging assay, the TBARS assay in brain homogenates, the FRAP assay of ferric iron (Fe^3+^) to ferrous iron (Fe^2+^), and the system β-carotene/linoleic acid based on the inhibition of β-carotene bleaching in the presence of linoleic acid radicals. The IC_50_ in the β-carotene/linoleic acid assay was achieved at a value of 0.90 mg/mL, in the DPPH radical scavenging assay, the value obtained was 0.69 mg/mL, the TBARS assay yielded a value of 0.43 mg/mL, and in the FRAP assay, the lowest value was obtained at 0.23 mg/mL [13].

As Asensio-S.-Manzanera et al. [33] reported, the DPPH and FRAP assays were used to evaluate the antioxidant activity on dry plant extracts of *T. mastichina* and dry residues after hydrodistillation. In the dry plant extracts, a scavenging effect was observed through the assays of DPPH (EC_50_ 0.59–1.78 mg/mL) and FRAP (EC_50_ 0.77–2.05 mg/mL). While for the hydrodistilled residue, the EC_50_ values were higher, showing less antioxidant activity. These results could be related to the higher phenol content in the dry extracts compared with the hydrodistilled residue, indicating that a considerable amount of antioxidants were retained in the remaining hydrodistilled water and *T. mastichina* essential oil.

Albano et al. [14], evaluated the antioxidant activity of *T. mastichina* essential oil and decoction water extract. The DPPH method was used with the essential oil and decoction water extract, while the scavenging activity of the superoxide anion radical was only used successfully with decoction water extract. For the DPPH method, the IC_50_ of the essential oil was much higher (6706.8 μg/mL) than that of the extract (4.2 μg/mL). The evaluation of the antioxidant activity by the superoxide anion scavenging activity method in the extract revealed that the IC_50_ of *T. mastichina* was 14.8 μg/mL. In this study, no correlation was detected between total phenols and removal of superoxide anion radicals. 

The antioxidant activity of *T. mastichina* ethanolic extracts, obtained by the *Soxhlet* system or ultrasound method, was also evaluated through the β-carotene/linoleate model system, FRAP, DPPH radical scavenging, and iron and copper ion chelation. In general, good antioxidant activities were obtained; in particular, for the *T. mastichina* extract obtained by Soxhlet extraction, which was even comparable to the antioxidant standard red grape pomace [51]. 

In a study conducted by Delgado et al. [37], the DPPH and FRAP assays of the methanolic extracts and essential oils from 20 different populations were examined. All methanolic extracts and essential oils presented a similar concentration-dependent pattern, increasing the scavenging activity against DPPH with the increase in concentration. However, much higher DPPH radical scavenging abilities were obtained with the methanolic extracts than with the essential oils. The results of the inhibition of DPPH radicals ranged between 86.6% and 93.9% when the highest methanolic extract concentration was used. In contrast, the essential oils of *T. mastichina* presented a much lower DPPH radical scavenging activity, varying between 30.8% and 57.7%, even when the highest concentration was used. Relative to the reductive power assay, it was only determined for the methanolic extracts because it was not possible to perform the assay correctly with the essential oils. Interestingly, in this study, the authors tried to relate the antioxidant activity to their chemical composition. In fact, *T. mastichina* methanolic extracts were rich in rosmarinic acid, which is a polyphenol carboxylic acid, known for possessing antioxidant activity, whereas the essential oils had low contents of thymol, among others, that could explain the low antioxidant activity.

The antioxidant activity of 14 populations of *T. mastichina* methanolic extracts grown in an experimental plot was analyzed by DPPH and FRAP assays to determine antioxidant activity. Population means for DPPH activity ranges were 44–98 mg TE/g dry weight (DW), while FRAP antioxidant capacity was 52–115 mg of Trolox equivalents (TE)/g DW. In general, populations with high DPPH free radical scavenging assay also showed high FRAP antioxidant activity and vice-versa. In this study, the rosmarinic acid contributed mainly to the FRAP antioxidant capacity and total phenolic content, while the unknown compound (peak 3) contributed mainly to the DPPH assay. This study showed high intrapopulation variability and, above all, high interpopulation variability [9].

Chitosan edible films incorporated with *T. mastichina* essential oil (concentrations of 1% and 2%) were tested by DPPH and FRAP methods. The DPPH method demonstrated lower values (0.29 and 0.44 mg/g, respectively) compared to the FRAP method (2.21 and 3.99 mg/g, respectively) [54]. These investigators also evaluated the antioxidant activity but under different conditions. The concentrations used ranged from 0.23 to 30 mg/mL of *T. mastichina* essential oil for DPPH methods, 2,2′-azino-bis(3-ethylbenzothiazoline-6-sulfonic acid) (ABTS) radical cation scavenging activity assay, and FRAP assay, while the concentrations used ranged from 0.15 to 20 mg/mL of *T. mastichina* essential oil for the ferrous ion-chelating ability assay. The DPPH method showed the lowest IC_50_ values (3.11 mg/mL), followed by the ABTS radical scavenging method (3.73 mg/mL), followed by the ferrous ion-chelating activity (9.61 mg/mL). Relative to the FRAP assay, the results were 19.26 mg TE/mL. *T. mastichina* essential oil can be used in general by the food and pharmaceutical industry as a potential natural additive, replacing or reducing the use of chemical substances, since it has antioxidant properties [55]. 

In the study conducted by Delgado-Adámez et al. [44], the antioxidant activity of *T. mastichina* essential oil showed an antioxidant activity lower than 4 g Trolox/L determined by the ABTS radical method.

In another study, the antioxidant activities of *T. mastichina* essential oil and aqueous extract were evaluated. The essential oil in the β-carotene/linoleic acid method presented an IC_50_ of 0.622 mg/mL, while the aqueous extract presented values of 0.017 mg/mL activity. In the radical DPPH method, the IC_50_ values were 9.052 and 0.104 mg/mL for the essential oil and aqueous extract, respectively. Finally, for the FRAP test, the IC_50_ values presented the same pattern (18.687 and 0.109 mg/mL for the essential oil and aqueous extract, respectively). Thus, it was clear that the extract showed higher antioxidant activity than the essential oil due to the high content of phenolic compounds in extracts [43]. These results are in accordance with the study of Albano et al. [14], in which the extracts also showed higher scavenging ability. In a recent study from the same investigators, the antioxidant activity of *T. mastichina* essential oil was corroborated through the DPPH radical method, FRAP assay, and β-carotene/linoleic acid system. This activity could be related to the high content of oxygenated monoterpenes (85.9%), which act as radical scavengers and ferric reducers with high activity to protect the lipid substrate [2].

The antioxidant activities of *T. mastichina* essential oils have been evaluated using several complementary methods: oxygen radical absorbance capacity (ORAC) that measures the antioxidant capacity against peroxyl radicals, ABTS, DPPH, TBARS, and chelating power. The results obtained in the different methods were the following: ORAC method (163.5–735.1 mgTE/g), ABTS method (0.8–4.3 mg TE/g), DPPH assay (53.5–76.1 mg TE/kg), TBARS method (0.9–1.2 mg BHT equivalents (BHTE)/g), and chelating power (0.6–1.6 mg ethylenediaminetetraacetic acid equivalents (EDTAE)/g). In general, the different antioxidant activities were related to the individual constituents that were also tested in these assays [4].

Taghouti et al. [10] showed that the *T. mastichina* hydroethanolic extract presented a significantly higher scavenging activity of the ABTS radical cation (≈1.48 mmol TE/g extract) than the aqueous decoction extract (≈0.96 mmol TE/g extract). For the hydroxyl radical and nitric oxide radical scavenging assays, both extracts showed a similar capacity for scavenging. These screening assays showed that *T. mastichina* extracts may be a potential source of phenolic compounds with relevant antioxidant activities, inclusively using the decoction that is the traditional method of consumption.

### 4.3. Anticancer Activity

Dichloromethane and ethanol extracts from the aerial parts of *T. mastichina* were evaluated regarding anticancer activity on the colon cancer cell line HCT, presenting IC_50_ values of 2.8 and 12 μg/mL, respectively. Additionally, one constituent of the extract, ursolic acid, was found to have an IC_50_ value of 6.8 μg/mL, while the other compounds in the extract were inactive with an IC_50_ > 20 μg/mL. A mixture of oleanolic and ursolic acid showed higher cytotoxicity than pure ursolic acid (IC_50_ of 2.8 μg/mL). The presence of these constituents identified by colon cancer cytotoxicity-guided activity indicates that *T. mastichina* extracts may have a protective effect against colon cancer [52]. 

The cytotoxic effects of *T. mastichina* essential oil were evaluated on human epithelioid cervix carcinoma (HeLa) and human histiocytic leukemia (U937) cell lines. A dose-dependent decrease in the survival of both tumor cell lines was observed after treatment with *T. mastichina* essential oil; this decrease was statistically significant at a concentration of 0.1% (*v*/*v*) *T. mastichina* essential oil [44].

The antiproliferative effect of *T. mastichina* essential oil against human breast carcinoma cell line MDA-MB-231 was also evaluated, showing an IC_50_ value of 108.5 μg/mL. From the literature, studies reported that antiproliferative activity could be related to 1,8-cineole content and are dependent on the monoterpenes content and their ability to affect oxidative stress [2]. It has been appointed that the preventive effect of essential oils on cancer disorders could be related to the promotion of cell cycle arrest, stimulating cell apoptosis and DNA repair mechanisms, inhibiting cancer cell proliferation, metastasis formation, and multidrug resistance, which makes them potential candidates for adjuvant anticancer therapeutic agents.

In a recent study, a *T. mastichina* aqueous decoction and hydroethanolic extract presented a dose and time-dependent inhibitory effect on the cell viability of a human colon adenocarcinoma (Caco-2) cell line and human hepatocellular carcinoma (HepG2) cell line. The hydroethanolic extract presented higher antiproliferative activity/cytotoxicity on Caco-2 cells (IC_50_: 71.18 and 51.30 μg/mL after 24 and 48 h of incubation, respectively) than the aqueous decoction extracts (IC_50_: 220.68 and 95.65 μg/mL after 24 and 48 h of incubation, respectively), which was correlated with its higher phenolic content. In addition, it should be noted that the Caco-2 cells were more sensitive than HepG2 cells, as it presented lower IC_50_ values for both extracts [10].

### 4.4. Antiviral Activity

*T. mastichina* essential oil was evaluated for its ability to reduce or eliminate the most emergent foodborne viruses in the food industry, evaluating its potential to inactivate two model nonenveloped viruses, a human norovirus surrogate, murine norovirus (MNV-1) with RNA genome, and a human adenovirus serotype 2 (HAdV-2) with DNA genome. However, no significant reduction of virus titers was observed when *T. mastichina* essential oil was used at different temperatures and times [59].

The influenza virus is associated with respiratory tract complications. Thus, in a study conducted by Choi [46], *T. mastichina* essential oil demonstrated interesting anti-influenza activity of reducing visible cytopathic effects of the A/WS/33 virus. This anti-influenza A/WS/33 activity of *T. mastichina* essential oil appeared to be associated with the constituent linalool. 

### 4.5. Insecticidal and Insect Repellent Activity

In recent years, plants have been identified for their insecticidal or larvicidal properties and used to control insect vectors offering an economically viable and ecofriendly approach. One of the studies was based on exposing *Spodoptera littoralis* larvae to *T. mastichina* essential oil and verifying larval mortality. The essential oil was highly toxic with a lethal concentration at 50% (LC_50_) of 19.3 mL/m^3^ when applied by fumigation. After topical application, *T. mastichina* essential oil was also highly toxic with a LC_50_ of 0.034 μL/larvae [60]. The same investigator also evaluated the topical and fumigant activity of essentials oils on the adult house fly (*Musca domestica* L.) and determined a topical LD_50_ of 33 μg/fly and fumigant LD_50_ of 7.3 μg/cm^3^ [61].

### 4.6. Anti-Alzheimer Activity

Alzheimer’s disease is characterized by the loss of cholinergic neurons, leading to the progressive reduction of acetylcholine in the brain and cognitive impairment. Inhibition of acetylcholinesterase (AChE) and butyrylcholinesterase (BChE) has great potential in the treatment of Alzheimer’s disease and special focus has been directed to these targets.

Albano et al. [14] reported the AChE inhibition activity of *T. mastichina* essential oil for the first time, with IC_50_ values of 45.8 μg/mL related to the 1,8-cineole constituent. However, the assessment of decoction water extracts was not possible for AChE inhibition capacity. Aazza et al. [40] also reported AChE inhibition activity of *T. mastichina* essential oil but poor activity was observed (IC_50_ of 0.1 mg/mL), despite the fact that the main constituent was also 1,8-cineole. 

In another study, *T. mastichina* essential oil revealed a high ability to inhibit cholinesterase activity with an IC_50_ of 78.8 and 217.1 μg/mL for AChE and BChE, respectively. The aqueous extract also showed AChE activity, with IC_50_ values of 1003.6 and 779.1 μg/mL for AChE and BChE, respectively. These results suggest that the essential oils and extracts of this aromatic plant could be useful in the treatment of Alzheimer’s disease [43].

Finally, *T. mastichina* essential oil was also reported to have AChE inhibition activity with an IC_50_ of 57.5–117.2 μg/mL. These results support the possible use of *T. mastichina* essential oils as an aid in the treatment of Alzheimer’s disease or in its prevention for people with family precedents [4].

### 4.7. Anti-Inflammatory Activity

The 5-lipoxygenase (5-LOX) activity is an assay used to evaluate both anti-inflammatory and antioxidant activities. 

Albano and Miguel [50] evaluated the 5-LOX activity of deodorized (divided into three fractions: the first one suspended in methanol; the second fractionated with water and chloroform; the third with chloroform), organic (diethyl ether, ethyl ether, and *n*-butanol), and aqueous extracts of *T. mastichina*. This study reported that lower IC_50_ values (12.2 μg/mL) were obtained for the water-insoluble deodorized chloroformic fraction of deodorized extract. In contrast, a higher IC_50_ was obtained using diethyl ether, with an IC_50_ of 62.5 μg/mL. Finally, for the deodorized fraction suspended in methanol and the aqueous extract, it was not possible to determine the IC_50_. For most of these extracts in the 5-LOX assay, there was a correlation between phenol content and IC_50_ values, meaning that a higher phenolic content in the extract resulted in a lower 5-LOX activity.

The *T. mastichina* essential oil revealed anti-inflammatory activity, being able to inhibit 5-LOX, with an IC_50_ of 1084.5 μg/mL, whereas the extracts showed an IC_50_ of 66.7 μg/mL. The constituents of the *T. mastichina* essential oil have also been shown to be as effective as 5-LOX inhibitors, such as 1,8-cineole [14]. In another study, a similar IC_50_ of 0.73 mg/mL for the *T. mastichina* essential oil was also reported [40]. 

*T. mastichina* essential oil was reported to present 5-LOX inhibition activity that was expressed as the degree of inhibition (DI (%)). *T. mastichina* essential oil, at a concentration of 150 μg/mL, showed a DI between 40.8% and 56.7% [4].

### 4.8. α-Amylase and α-Glucosidase Activity

Inhibition of α-amylase and α-glucosidase activity was also studied for *T. mastichina* essential oil, with reported IC_50_ values of 4.6 and 0.1 mg/mL, respectively [40]. The inhibition of these enzymes could result in the slow and prolonged release of glucose into circulation and, consequently, the retardation of sudden hyperglycemia after the consumption of a meal.

## 5. Conclusions

*T. mastichina* essential oil was obtained mainly by hydrodistillation, consisting mainly of 1,8-cineole (eucalyptol), linalool, limonene, camphor, borneol, and α-terpineol, as well as other volatile compounds. Conversely, despite being lesser studied, *T. mastichina* extracts using different solvents were also characterized, being composed of 2-methoxysalicylic acid, 3-methoxysalicylic acid apigenin, caffeic acid, chlorogenic acid, kaempferol, luteolin, quercetin, rosmarinic acid, sakuranetin, sterubin, salvianolic acid derivatives, and hexoside and glycoside derivatives, among other constituents.

*T. mastichina* has been traditionally used as a flavoring for food and in the treatment of health conditions due to its antiseptic, digestive, antirheumatic, and antitussive effects. Regarding the biological activities reported in different studies, *T. mastichina* essential oil and/or extracts also have antibacterial, antifungal, antioxidant, insecticide, repellent, antiviral, anti-Alzheimer, and anti-inflammatory activities. The antibacterial and antifungal activities of *T. mastichina* are an important characteristic for the use of these plants for production as natural antimicrobial agents that could be used as preservatives against diverse Gram-positive and negative bacteria and fungi. In addition, the antioxidant activity of *T. mastichina* was also largely explored through different assays, representing an interesting alternative to synthetic antioxidants. Although little attention has been paid to other activities, such as insecticide, repellent, antiviral, anti-Alzheimer, and anti-inflammatory activities, *T. mastichina* also showed interesting potential for these activities. In some studies, these effects were related to the composition and were tested to understand if some compounds were primarily responsible for the observed activity.

In conclusion, attending to its traditional use and reported biological activities, *T. mastichina* essential oil and/or extracts could present a noteworthy role as preservatives and salt substitutes in food industries, as perfumes in cosmetic industry, and as sources of bioactive compounds for pharmaceutical industries.

## Figures and Tables

**Table 1:** Obtention features and characterization of the *Thymus mastichina* essential oil and its main constituents (equal or higher than 5%).

Plant Material (Growth Phase)	Period of Year	Source	Yield	Major Constituents	Reference
Flowering branches	May–July	Trás-os-Montes; Beira Alta; Beira Baixa; Estremadura; Ribatejo; Alto Alentejo; Algarve (Portugal)	2.2% (*v*/*w*)	1,8-cineole (53.3%); linalool (5.5%)	[15]
Leaves	December; May	Nave do Barão, Algarve (Portugal)	-	1,8-cineole (46.29%); camphor (10.77%); camphene (6.31%); α-pinene (5.23%)	[16]
Leaves	-	Vadofresno, Córdoba (Spain)	-	1,8-cineole (24.81%)	[17]
				1,8-cineole (18.87%)	
Leaves and flowers	December; May	Algarve (Portugal)	-	1,8-cineole (46.3–50.4%); camphor (9.6–10.8%); camphene (5.0–6.3%); α-pinene (4.0–5.3%);	[18]
Leaves	-	S. Brás de Alportel, Algarve (Portugal)	0.4–0.9% (*v*/*w*)	1,8-cineole (50.2–61.0%); camphor (7.6–10,1%); δ-terpineol (6.5–9.7%); camphene (4.4–6.1%)	[19]
Flowers			1.6–2.2% (*v*/*w*)	1,8-cineole (46.7–50.2%); δ-terpineol (5.9–8.2%)	
Aerial parts	December; May; June; October January; May, June; October (2 years)	S. Brás de Alportel, Algarve (Portugal)	-	1,8-cineole (42.1–50.43%); camphor (7.4–11.5%); camphene (3.1–6.3%); α-terpineol (3.4–5.7%); *trans*-sabinene hydrate (0.2–5.6%); α-pinene (3.1–5.3%)	[20]
Flowers (full flowering phase)	May	S. Brás de Alportel, Algarve, Sotavento (Portugal)	-	1,8-cineole (46.9%); camphor (6.7%); α-terpineol (5.2%)	[21]
Leaves (full flowering phase)				1,8-cineole (42.4%); camphor (7.7%); borneol (6.8%); α-terpineol (6.1%)	
Aerial parts (beginning of flowering phase)		Sesimbra, Estremadura (Portugal)	-	Chemotype A (aerial parts): linalool (44.4%); 1,8-cineole (37.4%) Chemotype B (aerial parts): linalool (61.4%); camphor (5.3%)	
Aerial parts (flowering phase)	-	Algarve (Portugal)	-	1,8-cineole (45.3%); camphor (8.5%); camphene (6.6%); α-pinene (5.4%); limonene (5.2%); borneol (5.0%)	[22]
		Estremadura (Portugal)		linalool (52.3%); 1,8-cineole (9.6%); limonene (6.4%); p-cymene (6.2%)	
Aerial parts	October; January; April; June	Nave do Barão, Algarve (Portugal)	0.7–3.6% (*v*/*w*)	1,8-cineole (45.1–58.6%); camphor (5.5–8.9%); α-pinene (4.6–6.8%); camphene (4.3–6.0%)	[23]
Aerial parts (vegetative phase and flowering phase)	October; May	Sesimbra (Portugal)	0.7–2.7% (*v*/*w*)	linalool (58.7–69%); 1,8-cineole (1.1–10.8%); elemol (0.9–6.6%); camphor (2.4–5.3%)	[24]
Aerial parts	January	Direção Regional de Agricultura de Trás-os-Montes (Portugal)	1.3% (*v*/*w*)	1,8-cineole (57.8%); limonene (10.8%)	[25]
Aerial parts	-	Direção Regional de Agricultura de Trás-os-Montes, Mirandela (Portugal)	-	1,8-cineole (67.4%)	[26]
Leaves	June	Sesimbra (Portugal)	2.2%	linalool (68.5%); 1,8-cineole (9.4%)	[8]
Flowers			2.6%	linalool (73.5%); 1,8-cineole (10.2%)	
Leaves		Arrábida (Portugal)	1.7%	1,8-cineole (69.2%); linalool (6.3%)	
Flowers			3.5%	1,8-cineole (54.6%); linalool (13.7%)	
Leaves		Mértola (Portugal)	2.0%	1,8-cineole (44.2%)	
Flowers			3.0%	1,8-cineole (39.4%); linalool (8.1%)	
Leaves		S. Brás de Alportel (Portugal)	1.4%	1,8-cineole (49.7%)	
Flowers			2.0%	1,8-cineole (48.5%)	
Aerial parts (flowering phase)		Mirandela (Portugal)	2.4% (*v*/*w*)	1,8-cineole (64.1%); α-terpineol (5.6%)	[27]
Aerial parts (vegetative phase)	January	Algarve (Portugal)	2.3% (*v*/*w*)	1,8-cineole (49.4%); limonene (9.3%)	[28]
Aerial parts	-	Córdoba (Spain)	-	1,8-cineole (45.67%); linalool (27.88%)	[29]
Aerial parts	-	Mértola (Portugal)		1,8-cineole (61.0%)	[30]
		Vila Real de Santo António (Portugal)	1.0–1.3% (*v*/*w*)	1,8-cineole (49.4%)	
		Sesimbra (Portugal)		linalool (39.7%); 1,8-cineole (9.6%)	
Plants (flowering phase)	-	Direção Regional de Agricultura e Pescas do Algarve (Portugal)	-	1,8-cineole (41.0%); β-pinene + trans-sabinene (7.0%); camphor (6.9%); borneol (6.5%); α-pinene (6.0%); camphene (5.5%)	[31]
Aerial parts (flowering phase)	June	Direção Regional de Agricultura e Pescas do Algarve (Portugal)	4% (*w*/*w*)	1,8-cineole (44%); camphor (10%); borneol (7%); camphene (7%); α-pinene (6%); α-terpineol (5%)	[32]
Plant (flowering phase)	Summer	Tordesillas, Valladolid; Truchas, Peradoones, Carrocera, Boñar, León; Almazán, Soria; Riaza, Villacastín, Segovia; Serranilos, Avila; Saldaña, Palencia (Spain)	3.40–6.90%	-	[33]
Aerial parts	-	Direção Regional de Agricultura e Pescas do Algarve (Portugal)	6.3% (*w*/*w*)	1,8-cineole (49.4%); α-pinene (7.0%); camphene (6.9%); camphor (5.8%); β-pinene (5.3%)	[14]
Whole plants	-	Barcelona (Spain)	-	1,8-cineole (52.57%); linalool (12.78%)	[34]
Commercial samples: leaves, stem, and flowers	-	Esencias Martinez Lozano, Murcia (Spain)	-	1,8-cineole (51.94%); linalool (19.90%)	[35]
Aerial parts (vegetative phase)	-	Coimbra (Portugal)	1.17% (*v*/*w*)	1,8-cineole (46%); limonene (23%)	[36]
Aerial parts (flowering phase)	June–July	Béjar, Valdemierque, Mozarbez, Golpejas, Salamanca; Carrocera, Boñar, Truchas, Peranzanes, León; Salas de los Infante, Lerma, Oña, Burgos; Villacastin, Riaza, Coca, Prádena, Segovia; Vinuesa, Aldealpozo, Almazán, Langa de Duero, Soria (Spain)	2.27–6.48% (*v*/*w*)	1,8-cineole (56.80–69.60%); linalool (0.62–15.7%); α-terpineol (2.07–5.99%); β-pinene (1.72–5.63%); limonene (1.07–5.10%)	[37]
Aerial parts	-	Vila Chã (Portugal)	-	1,8-cineole (47.4%); thymol (13.7%); p-cymene (9.7%); γ-terpinene (7.3%)	[38]
Flowers and leaves	June	Carrocera, Léon (Spain)		1,8-cineole + limonene (61.6%); linalool (6%); β-pinene (5.7%)	[39]
			-	1,8-cineole + limonene (69.3%)	
				1,8-cineole + limonene (64%)	
Plants (flowering phase)	-	Algarve (Portugal)	1.2% (*v*/*w*)	1,8-cineole (52.8%); α-pinene (7.2%); camphene (7.2%); camphor (7.2%)	[40]
Plants grown in vitro (all parts except roots)	-	Urbino (Italy)	0.56% (*v*/*w*)	1,8-cineole (55.6%); linalool (24.5%); β-pinene (5.9%)	[41]
Commercial samples		Planalto Dourado^TM^; Freixedas (Portugal)	-	1.8-cineole (49.94%); linalool (5.66%); α-terpineol (5.59%); β-pinene (5.54%)	[42]
Aerial parts	-	Évora, Alentejo (Portugal)	1.1% (*v*/*w*)	1,8-cineole (72.0%); α-terpineol (9.0%)	[43]
Flowers		Badajoz (Spain)	-	limonene + 1,8-cineole (71.82%); β-myrcene (9.81%); α-terpineol (5.32%); camphene (5.15%)	[44]
Fruits				limonene + 1,8-cineole (78.37%); β-myrcene (5.69%); α-terpineol (5.05%)	
Leaves	Summer	Alentejo (Portugal)	-	1,8-cineole (74.2%); α-terpenyl acetate (7.9%)	[45]
Leaves	-	UNIQ F&F Co., Ltd. (Seoul, Korea)	-	β-pinene (5.81%); 1,8-cineole (64.61%); linalool (15.28%)	[46]
Aerial parts	July	Murcia (Spain)	1.8–3.6% (*v*/*w*)	1,8-cineole (38.8–74.0%); linalool (13.3–42.7%)	[4]
Leaves and stem	-	Ciudad Real (Spain)	-	1,8-cineole (43.26%); linalool (36.72%); linalyl acetate (5.58%)	[47]
Commercial samples	-	Ervitas Catitas (Portugal)		1,8-cineole (55.9%); β-pinene (10.8%)	[48]
Aerial parts		Évora, Alentejo (Portugal)	1.06% (*v*/*w*)	1.8-cineole (71.2%); α-terpineol (9.7%)	[2]

**Table 2:** Antibacterial and antifungal activity of *Thymus mastichina* essential oils and extracts.

Origin	Micro-Organisms	Species	Measured Response and Results Obtained						References
S. Brás de Alportel, Algarve, Sotavento (Portugal)			Diameter of the zone of inhibition (mm), including the diameter of the disc (6 mm)						[21]
			Flower		Leaf				
	Gram-negative bacteria	*Escherichia coli*	8.0		14.0				
		*Proteus mirabilis*	7.0		7.3				
		*Salmonella* subsp.	8.0		8.7				
	Gram-positive bacteria	*Staphylococcus aureus*	13.7		15.7				
		*Listeria monocytogenes EGD*	9.7		12.3				
	Fungus	*Candida albicans*	10.0		11.0				
Sesimbra, Estremadura (Portugal)			Chemotype A		Chemotype B				
	Gram-negative bacteria	*Escherichia coli*	7.5		10.6				
		*Proteus mirabilis*	7.5		10.0				
		*Salmonella* subsp.	6.3		7.0				
	Gram-positive bacteria	*Staphylococcus aureus*	13.3		9.6				
		*Listeria monocytogenes EGD*	ND		11.0				
	Fungus	*Candida albicans*	10.6		13.6				
Direção Regional de Agricultura de Trás-os-Montes, Mirandela (Portugal)			MIC (μL/mL)		MLC (μL/mL)				[26]
	Fungi	*Candida albicans*	2.5		2.5				
		*Candida albicans*	1.25–2.5		2.5				
		*Candida albicans*	2.5		5.0				
		*Candida tropicalis*	2.5–5.0		5.0				
		*Candida tropicalis*	5.0–10.0		5.0				
		*Candida glabrata*	1.25–2.5		5.0				
		*Candida glabrata*	2.5		5.0				
		*Candida krusei*	1.25–2.5		2.5				
		*Candida guilhermondii*	1.25		1.25				
		*Candida parapsilosis*	2.5–5.0		5.0				
Córdoba (Spain)			MIC (%, *v*/*v*)						[29]
	Gram-negative bacteria	*Escherichia coli*—origin in poultry	4						
		*Salmonella enteritidis*—origin in poultry	4						
		*Salmonella essen*—origin in poultry	4						
		*Escherichia coli (ETEC)*—origin in pig	4						
		*Salmonella choleraesuis*—origin in pig	4						
		*Salmonella typhimurium*—origin in pig	4						
Barcelona (Spain)			Area of the inhibition zone (mm^2^) excluding the film area						[34]
			6%	7%	8%			9%	
	Gram-positive bacteria	*Listeria innocua*	NA ^a^	0.79 ^a^	0.79 ^a^			NF ^a^	
		Methicillin-resistant *Staphylococcus aureus*	NA ^a^	NA ^a^	NA ^a^			NF ^a^	
	Gram-negative bacteria	*Salmonella enteritidis*	NA ^a^	NA ^a^	NA ^a^			NF ^a^	
		*Pseudomona fragi*	NA ^a^	NA ^a^	NA ^a^			NF ^a^	
Monteloeder, SL (Elche, Spain)			MIC microdilution technique (μg/mL)		MIC dilution technique (μg/mL)		MBC broth dilution techniques (μg/mL)		[53]
			After 24 h	After 48 h	After 24 h	After 48 h	Microdilution	Tube dilution	
	Gram-negative bacteria	*Escherichia coli*	12,800 ^b^	25,600 ^b^	12,800 ^b^	25,600 ^b^	51,200 ^b^	51,200 ^b^	
		*Salmonella enterica*	6400 ^b^	12,800 ^b^	12,800 ^b^	25,600 ^b^	25,600 ^b^	51,200 ^b^	
		*Enterobacter aerogenes*	12,800 ^b^	51,200 ^b^	51,200 ^b^	102,400 ^b^	51,200 ^b^	102,400 ^b^	
	Gram-positive bacteria	*Bacillus cereus*	1600 ^b^	3200 ^b^	3200 ^b^	3200 ^b^	6400 ^b^	6400 ^b^	
		Methicillin-resistant *Staphylococcus aureus*	400 ^b^	800 ^b^	800 ^b^	1600 ^b^	1600 ^b^	3200 ^b^	
Esencias Martinez Lozano (Murcia, Spain)			Diameter of the inhibition zone (mm) including disc diameter (9 mm)				MIC (μL/mL)		[35]
	Gram-positive bacteria	*Listeria innocua*	26.83				3.75		
	Gram-negative bacteria	*Serratia marcescens*	12.36				7.5		
		*Pseudomonas fragi*	11.68				3.75		
		*Pseudomonas fluorescens*	9.0				3.75		
		*Aeromonas hydrophila*	11.29				3.75		
		*Shewanella putrefaciens*	9.0				3.75		
		*Achromobacter denitrificans*	10.69				3.75		
		*Enterobacter amnigenus*	12.51				7.5		
		*Enterobacter gergoviae*	12.14				7.5		
		*Alcaligenes faecalis*	23.50				3.75		
Esencias Martinez Lozano (Murcia, Spain)			Diameter of the inhibition zone (mm) including disc diameter (10 mm)						[54]
			1%				2%		
	Gram-positive bacteria	*Listeria innocua*	17.92 ^c^				25.51 ^c^		
	Gram-negative bacteria	*Serratia marcescens*	21.15 ^c^				32.36 ^c^		
		*Enterobacter amnigenus*	NA ^c^				NA ^c^		
		*Alcaligenes faecalis*	18.42 ^c^				28.29 ^c^		
Esencias Martinez Lozano (Murcia, Spain)			Diameter of the inhibition zone (mm) including disc diameter (9 mm)						[55]
			Minced beef		Cooked ham		Dry-cured sausage		
	Gram-positive bacteria	*Listeria innocua*	34.98		15.23		19.45		
	Gram-negative bacteria	*Achromobacter denitrificans*	11.29		13.29		15.87		
		*Alcaligenes faecalis*	16.91		15.34		16.03		
		*Aeromonas hydrophila*	14.7		12.13		24.94		
		*Enterobacter amnigenus*	10.97		10.69		17.31		
		*Enterobacter gergoviae*	10.82		13.81		9		
		*Pseudomonas fluorescens*	12.07		12.86		16.7		
		*Pseudomonas fragi*	11.61		11.78		14.19		
		*Serratia marcescens*	11.84		12.69		11.49		
		*Shewanella putrefaciens*	13.09		14.34		15.82		
			MIC (μg/mL)		MFC (mg/mL)				[41]
Urbino (Italy)	Fungi	*Fusarium culmorum*	1500		2				
		*Fusarium graminearum*	1500		2				
		*Fusarium poae*	1500		2				
		*Fusarium avenaceum*	1500		2				
		*Fusarium equiseti*	2100		2.4				
		*Fusarium semitectum*	2000		2.4				
		*Fusarium sporotrichoides*	2000		2.4				
		*Fusarium nivale*	2000		2.4				
Alentejo (Portugal)			MIC (mg/mL)		MBC (mg/mL)				[45]
	Gram-positive bacteria	Methicillin-sensitive *Staphylococcus aureus*	20		40				
		*Bacillus subtilis*	15		30				
	Gram-negative bacteria	*Escherichia coli*	15		30				
		*Pseudomonas aeruginosa*	20		70				
Ciudad Real (Spain)			ED_50_						[47]
	Fungi	*Botrytis cinerea*	-						
		*Sclerotinia sclerotiorum*	14.87						
		*Fusarium oxysporum*	58.0						
		*Phytophthora parasitica*	22.0						
		*Alternaria brassicae*	>100						
		*Cladobotryum mycophilum*	14.1						
		*Trichoderma agressivum*	-						
Murcia (Spain)			MIC (mg/mL)		MBC (mg/mL)				[4]
	Gram-negative bacteria	*Escherichia coli*	2.3–9.4		2.3–9.4				
	Gram-positive bacteria	Methicillin-sensitive *Staphylococcus aureus*	2.3–4.7		4.6–4.7				
	Fungus	*Candida albicans*	2.3–4.7		2.3–4.7				
Ervitas Catitas (Portugal)			Inhibition growth zone (mm)		MIC (μg/mL)				[48]
	Gram-positive bacteria	Methicillin-sensitive *Staphylococcus aureus* (isolates)	9.0–11.8		500–4000 (or higher)				
		*Staphylococcus epidermidis* (isolates)	ND; 9.0–13.8		4000–4000 (or higher)				
Évora, Alentejo (Portugal)			Inhibition growth zone (mm)		MIC (μL/mL)				[2]
	Gram-positive bacteria	*Methicillin-sensitive* *Staphylococcus aureus*	19		>2				
		*Staphylococcus epidermidis*	21		>2				
		*Enterococcus faecalis*	21		>2				
	Gram-negative bacteria	*Escherichia coli*	11		>2				
		*Morganella morganii*	17		1.1				
		*Proteus mirabilis*	9		0.5				
		*Salmonella enteritidis*	11		0.1				
		*Salmonella typhimurium*	8		>2				
		*Pseudomonas aeruginosa*	17		1.1				

ED_50_ (effective dose 50), concentration that inhibits mycelial growth by 50%; MBC, minimum bactericidal concentration; MFC, minimal fungicidal concentration; MIC, minimum inhibitory concentration; MLC, minimum lethal concentration; NA, not active; ND, not determined; NF, no film formed. ^a^ Whey protein isolate films incorporated with *T. mastichina* essential oil ^b^ Mixture of *Rosmarinus officinalis*, *Salvia lavandulifolia*, and *T. mastichina* and chitosan ^c^ Chitosan edible film disks incorporated with *T. mastichina* essential oil.

## Supplementary Materials

The following are available online at https://www.mdpi.com/1424-8247/13/12/479/s1, Table S1: Obtention features and characterization of *Thymus mastichina* extracts and its main constituents and total phenolic and flavonoids contents, Figure S1: Chemical structures of majority of the identified constituents of the *Thymus mastichina* extracts (flavonoids, phenolic acids, phenolic terpene, steroid, triterpenoids and xanthophyll).
