# Supplementary material for: Thymus mastichina: Composition and Biological Properties with a Focus on Antimicrobial Activity
Source: Pharmaceuticals (Basel). 2020 Dec 19;13(12):479. doi: 10.3390/ph13120479 (PMC7766293; doi:10.3390/ph13120479)
Supplement: Supplementary file 1 [file pharmaceuticals-13-00479-s001.pdf]

Supplementary material

Thymus mastichina: Composition and Biological Properties with a Focus on Antimicrobial Activity

Márcio Rodrigues [1,2,3]*, Ana Clara Lopes [1], Filipa Vaz [1], Melanie Filipe [1], Gilberto Alves [3], Maximiano P. Ribeiro [1,2,3], Paula Coutinho [1,2,3] * and André R. T. S. Araujo [1,2,4] *

[1] School of Health Sciences, Polytechnic Institute of Guarda, Rua da Cadeia, 6300-035 Guarda, Portugal; claralopes28@gmail.com (A.C.L.); filipa.a.c.vaz@hotmail.com (F.V.); melaniemfilipe@gmail.com (M.F.); mribeiro@ipg.pt (M.P.R.)

[2] Research Unit for Inland Development (UDI), Polytechnic Institute of Guarda, Av. Dr. Francisco Sá Carneiro, 50, 6300-559 Guarda, Portugal

[3] CICS-UBI – Health Sciences Research Centre, University of Beira Interior, Av. Infante D. Henrique, 6200-506 Covilhã, Portugal

[4] LAQV/REQUIMTE, Department of Chemical Sciences, Faculty of Pharmacy, University of Porto, Rua Jorge Viterbo Ferreira, 228, 4050-313 Porto, Portugal

* Correspondence: marciorodrigues@ipg.pt (M.R); coutinho@ipg.pt (P.C.); andrearaujo@ipg.pt (A.R.T.S.A.) Tel.: +351-271-220-191

Table S1. Obtention features and characterization of *Thymus mastichina* extracts and its main constituents and total phenolic and flavonoids contents.

Plant material (growth phase)	Period of year	Source	Extraction	Yield	Total phenolic content	Flavonoids	Analytical system	Major constituents	References
Aerial parts (flowering phase)	June	Direção Regional de Agricultura e Pescas do Algarve (Portugal)	Remaining hydrodistillation-aqueous extract (water and chloroform)	0.5–9.7% (w/w)	-	-	-	-	[32]
			Hexane, Dichloromethane, and methanol extract	4.0–21.2% (w/w)	-	-	-	-	
Inflorescences	July	Bragança, Trás-os-Montes (Portugal)	Aqueous, ethanol/water (50:50, v/v) methanol extracts	21.61% (w/w)	47.11–165.29 mg GAE/g	3.18–83.85 mg CE/g	-	-	[13]

Aerial parts	-	Direção Regional de Agricultura e Pescas do Algarve (Portugal)	Remaining hydrodistillation-deodorized extract (methanol; water:chloroform; chloroform fractions)	5.33–22.01% (w/w)	0.78–21.38 mg GAE/mL	-	-	-	[50]
			Diethyl ether, ethyl acetate, *n*-butanol, and water extracts	0.24–1.56% (w/w)	2.23–26.28 mg GAE/mL	-	-	-	
Aerial parts	-	Direção Regional de Agricultura e Pescas do Algarve (Portugal)	Remaining hydrodistillation-aqueous extract (dimethyl sulfoxide: water, 3:1)	41.1%	22.9%	-	-	-	[14]
Aerial parts	-	Porto (Portugal); Murcia (Spain)	Hexane, dichloromethane and ethanol extracts	-	-	-	TLC plates/RP-18 silica gel columns; IR, 1D and 2D NMR and mass spectrometry	Sakuranetin; sterubin; oleanolic acid; ursolic acid; xanthophyll lutein; steroid β-sitosterol; rosmarinic acid; 6-hydroxyluteolin-7-O-glucopyranoside; 6- hydroxyapigenin-7-O-glucopyranoside	[52]
-	-	-	Soxhlet ethanolic extract	11.7% (w/w)	22.2% (w/w)	-	HPLC-ESI-MS	Hydroxy-cinnamoylquinic acid; quercetin glucoside; luteolin glucoside; rosmarinic acid;	[51]
			Ultrasound-assisted ethanolic extract	7.1% (w/w)	7.7% (w/w)	-			

								apigenin-7-O-glucoside; quercetin; luteolin derivative; naringenin; luteolin; carnosol; apigenin; kaempferol	
Aerial parts	Summer	Arévalo, El Barraco, Serranillos, Ávila; Villarcayo de Merindad de Castilla la Vieja, Oña, Lerma, Salas de los Infantes, Burgos; Villamañan, Carrizo, Truchas, Peranzanes, Vega de Espinareda, Páramo del Sil, Cabrillanes, Carrocera, Boñar, León; Saldaña, Guardo, Salinas de Pisuerga, Palencia; Vitigudino, Fuenteaguinaldo, Sequeros, Béjar, Valdemierque, Mozarbez, Villamayor, Golpejas, Salamanca; San Ildefonso/La Granja, Prádena, Riaza, Ayllón, Coca, Villacastín, Segovia; Burgo de Osma-Ciudad de Osma, Almazán, Aldealpozo,	Hydrodistilled residue	-	2.72–12.98 mg/g	-	HPLC-DAD	Apigenin; kaempferol; luteolin; 3-methoxysalicylic acid; rosmarinic acid	[12]

		Los Rábanos, Langa de Duero, Vinuesa, Soria; Tordesillas, Valladolid; Toro, Muelas del Pan, Fermoselle, Riofrío de Aliste, Benavente, Zamora (Spain)							
Aerial parts (flowering phase)	June-July	Béjar, Valdemierque, Mozarbez, Golpejas, Salamanca; Carrocera, Boñar, Truchas, Peranzanes, León; Salas de los Infante, Lerma, Oña, Burgos; Villacastin, Riaza, Coca, Prádena, Segovia; Vinuesa, Aldealpozo, Almazán, Langa de Duero, Soria (Spain)	Methanolic extract	-	2.90–9.15 mg GAE/g	-	HPLC-UV/visible	2-methoxysalicylic acid; apigenin; caffeic acid; kaempferol; luteolin; quercetin; rosmarinic acid	[37]
Flowers and leaves (beginning of flowering phase)	June-July	Almazán, Soria; Carrocera, Ponferrada, León; Casas de Lázaro, Lezuza, Albacete; Hontanar, Toledo; Lerma, Burgos; Moral de Calatrava, Ciudad Real; Riaza, Villacastín, Segovia; Saldana, Palencia; Serranillos, Ávila; Tordesillas, Valladolid; Toro, Zamora (Spain)	Ultrasound methanolic extract	-	6.8–56.4 mg CAE/g DW	-	HPLC-DAD	Rosmarinic acid; chlorogenic acid; luteolin; caffeic acid; luteolin glucoside	[9]

Aerial parts	-	Évora, Alentejo (Portugal)	Remaining hydrodistillation-aqueous extract	-	~80 mg GAE/g	~20 mg QE/g	-	-	[43]
Aerial parts	-	ERVITAL®, Plantas Aromáticas e Medicinais, Lda; Mezio (Portugal)	Aqueous decoction	9.32% (w/w)	134.76 mg CA/g	195.53 mg EC/g	HPLC-DAD and HPLC-ESI-MS^n	Caffeic acid; quercetin-O-hexoside; luteolin-O-hexoside; salvianolic acid B/E isomer 2; salvianolic acid A isomer; rosmarinic acid; salvianolic acid K; chrysoeriol-O-hexuronide	[10]
	-		Hydroethanolic extract	13.78% (w/w)	178.89 mg CA/g	184.45 mg EC/g		Quercetin-O-hexoside; luteolin-O-hexoside; salvianolic acid B/E isomer 2; salvianolic acid A isomer; rosmarinic acid; salvianolic acid K; salvianolic acid I; chrysoeriol-O-hexuronide	

CA/g, caffeic acid equivalents per gram; DW, dry weight; EC/g, catechin equivalents per gram; GAE/g, equivalents of gallic acid per gram; HPLC-DAD, high-performance liquid chromatography with diode array detection; HPLC-ESI-MS, high-performance liquid chromatography with electrospray ionization and mass spectrometry detection; HPLC-ESI-MS^n, high-performance liquid chromatography with electrospray ionization and tandem mass spectrometry detection; HPLC-UV/visible, high-performance liquid chromatography with ultraviolet/visible detection; IR, infrared; NMR, nuclear magnetic resonance; QE/g, quercetin equivalents per gram; TLC, thin layer chromatography.

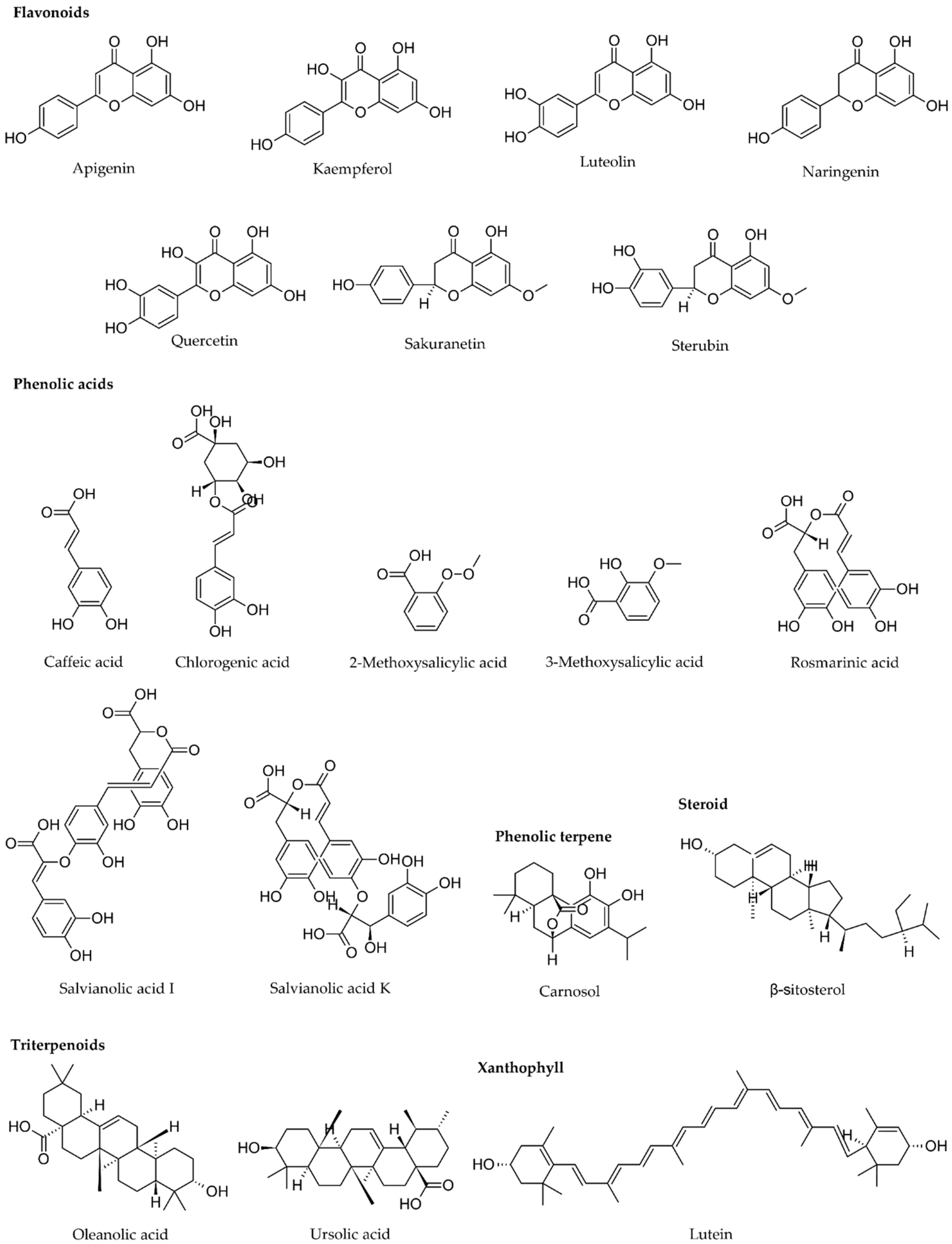

Figure S1. Chemical structures of majority of the identified constituents of the *Thymus mastichina* extracts (flavonoids, phenolic acids, phenolic terpene, steroid, triterpenoids and xanthophyll).
